# Determining Factors Affecting the Users’ Participation of Online Health Communities: An Integrated Framework of Social Capital and Social Support

**DOI:** 10.3389/fpsyg.2022.823523

**Published:** 2022-06-14

**Authors:** Xiu-Fu Tian, Run-Ze Wu

**Affiliations:** ^1^College of Business, Jiaxing University, Jiaxing, China; ^2^College of Economics, Jiaxing University, Jiaxing, China

**Keywords:** online health communities, users’ participation, social capital, social support, knowledge acquisition, knowledge contribution

## Abstract

As the national awareness of health keeps deepening, online health communities (OHCs) have achieved rapid development. Users’ participation is critically important to the sustainable development of OHCs. Nevertheless, users usually lack the motive for participation. Based on the social capital theory, this research examines factors influencing users’ participation in OHCs. The purpose of this research is to find out decisive factors that influence users’ participation in OHCs, enrich the understanding of users’ participation in OHCs, and help OHCs address the issue of sustainable development. The research model was empirically tested using 1277 responses from an online survey conducted in China. Data was analyzed using the structural equation modeling (SEM). We found informational support and emotional support to have significant direct effects over the structural capital, relational capital and cognitive capital of OHCs. Meanwhile, it is observed that relational capital and cognitive capital degree have a significant influence on knowledge acquisition and knowledge contribution of OHCs. For researchers this study provides a basis for further refinement of individual models of users’ participation. For practitioners, understanding the social capital is crucial to users’ knowledge acquisition and knowledge contribution that achieve high participation in OHCs.

## Introduction

As a country with a population of around 1.4 billion, China currently has around 400 million patients with chronic diseases. Management of chronic diseases outside the hospital exists as a tricky issue. Meanwhile, treatment of chronic diseases is a long-term process, which requires lifelong treatment, long-term medication and health management (Lai et al., 2021; Wang et al., 2021; Wu H. et al., 2021). Under the traditional medical care model, patients need to communicate with doctors about their disease in hospital. Because of difficulty in making an appointment with some doctors and the expensiveness of medical treatment, patients’ medical experience has been low and inefficient (Liu et al., 2021; Wu Y. et al., 2021). The problem should be mainly attributed to the inadequacy and uneven distribution of overall medical resources in China (Shao et al., 2016; Wang Q. et al., 2016; Li et al., 2020). Specifically, this problem is reflected as inadequacy of overall medical resources, including doctors, nurses, medical resources, and per capita disposable medical fees (Ye et al., 2019; Zheng et al., 2019; Xu et al., 2021).

With the development of information and technology, people can seek medical advice from the Internet (Ziebland and Wyke, 2012; Wu J.-J. et al., 2021; Yang et al., 2021). From the Internet, patients can search for information related to their need of medical health and consulting doctors or other patients online on health issues (Wu et al., 2018; Liu and Wang, 2021). Internet medical care can not only reasonably serve part of patients who do not have to seek consultation in hospitals offline, but also reduce the waste of medical resources and medical fees (Mo and Deng, 2015; Hossain, 2016). Moreover, the time cost for patients to receive medical treatment face to face is efficiently brought down, and the limited number of quality medical resources are left for those who are genuinely in need (Feng et al., 2021; Wang et al., 2021). Online health communities (OHCs) or online health communities exist as a community for health information exchange, where users can have health consultation, experience sharing, knowledge sharing and emotional sharing (Peng et al., 2019; Qiao et al., 2021). So far, a majority of OHCs have developed functions like health consulting, online socializing, themed recommendations, etc. These functions can help users realize long-term health management and establish a long-term communication system. Information sharing behaviors of users on OHCs community platforms can not only help improve the efficiency of users’ health information acquisition efficiency and alleviate the asymmetry of health information, but also help community operators increase the activity of community users, thus providing more comprehensive information services to satisfy users’ information sharing needs (Audrain-Pontevia and Menvielle, 2018). Kingod et al. (2016) and Petrič et al. (2017) thought that OHCs can create a supportive space for daily self-care of patients suffering from chronic diseases under effective support of doctors, and at the same time, OHCs can construct the offline relationship between patients and doctors and contribute to the harmony of the relationship between doctors and patients. Additionally, the rapid communication of information, suggestions or advice among OHC community members is conducive, since the OHCs usually deliver referral information related to diseases and health, suggestions, individual experience, etc. (Oh and Lee, 2012). Emotional sharing can facilitate the expression of sentiments, such as understanding, encouragement, sympathy and care among OHC members, through which the pressure and anxiety imposed on OHC members can be mitigated.

As a new platform for knowledge sharing and exchange in the modern medical field, OHCs ensures community members to contribute their role to efficient diffusion of medical knowledge *via* the information exchange (Jasmina et al., 2020; Mirzaei and Esmaeilzadeh, 2021). Not only can this help people deepen their knowledge of health, but also this can promote the transformation of the focus of the health concept from treatment to prevention, thus allowing people to have access to more medical and health care information and more reasonably pursue self-health management (Ma et al., 2021; Meng et al., 2021).

Characterized by user-generated content (UCG), OHCs requires deep participation of community members to accumulate mass data of health and medical care (Abedin et al., 2020; Lu and Zhang, 2021; Zhou, 2021). However, a majority of users remain an inactive status, who mainly acquire health knowledge and information from the community, lacking the motive and intention to share (Introne and Goggins, 2019; Liu S. et al., 2020). Nevertheless, current literatures generally focus on the information search and adoption stage of users’ participation. This has resulted in OHCs’ lack of theoretical guidance on how to protect users’ knowledge acquisition and knowledge contribution. On the other hand, OHCs is an information exchange and relation network made up of community members, including doctors, patients, family members and passers-by. Users’ behavioral decision-making is subject to the influence of not only community quality factors, such as system quality, information quality, service quality, but also structural, relational cognitive dimensions, which actually refer to social capital. For example, other users’ likes, comments, replies, number of followers, activeness and joint vision can all affect users’ participatory behaviors (Peng et al., 2019; Zhou, 2019b; Chen et al., 2020). Therefore, an in-depth research into factors influencing OHCs’ user participation is necessary to address the issue of how social capital can prompt users to adopt knowledge and information from OHCs and how users and other members can be encouraged to share knowledge and medical data.

Therefore, this research adopts social capital and its components to explore their influence on information adoption and sharing. At the same time, informational support and emotional support are introduced to the research model to improve the explaining capacity and applicability of this model. This research aims at examining how people’s assessment and experience of social capital affect their knowledge acquisition and knowledge contribution. To sum up, this research can not only help people acquire more medical care information and manage their own health more reasonably, but also provide a scientific basis for the sustainable development of OHCs.

The paper is structured as follows. In the next section we describe the concept of OHCs, literature review and theoretical background. Section “Research Model and Research Hypotheses” reports we propose the research model and hypotheses. Sections “Materials and Methods” and “Results” reports the instrument development, data collection process, data analysis, and results. In section “Discussion” we discusses these results. Then, we present the theoretical and practical implications in section “Theoretical and Practical Implications.” We conclude the paper by summarizing the limitations of the study and suggesting avenues for future research in section “Limitations.”

## Background and Literature Review

### Online Health Communities

In the narrow sense, OHCs can be divided by user types into online doctors’ communication communities and online doctor–patient communication communities (Zhou et al., 2019; Liu J. et al., 2020). In the broad sense, OHCs is mainly divided into professional health and medical care websites (Wu and Lu, 2017; Wu and Deng, 2019) represented by “Patients Like Me” and “Online Good octor,” health and medical care sections of large-scale social networks represented by Baidu Post Bar and Douban (Zhang et al., 2017b; Liu et al., 2018), and temporary chatting groups spontaneously organized by patients or enthusiasts of health knowledge (Oh et al., 2013; Li et al., 2019). The OHCs discussed in this paper tend to adopt the classification in the broad sense, which is a set of online communities that organize activities related to health information and aiming at providing a platform for users to express their opinions, share their experience, exchange their health information, offer or seek medical and health care information.

Management of chronic diseases after patients are discharged from hospital remains a tremendous challenge. Since the treatment of chronic diseases is a lengthy process, patients need long-term medication and health management control. OHCs are a relationship network established based on information sharing of community members, including doctors, patients, family members, passers-by, etc. This has led to a large amount of health and medical care data created, record or inferred by community members, which can promote the efficient diffusion and sharing of online medical health knowledge. Thereby, patients can have an easier access to health information. This can not only smoothen the information interaction between patients and doctors, but also improve the patient–doctor relationship, and continuously attract patients to receive treatment in accordance with doctor’s advice (Kingod et al., 2016; Petrič et al., 2017; Audrain-Pontevia and Menvielle, 2018).

A main challenge facing every online community is the user activity, that is, how to increase the information search frequency and promote the information sharing between users and other members (Chen et al., 2020). Therefore, to encourage users to use community information actively and provide knowledge sharing is essential to the survival and development of OHCs (Mirzaei and Esmaeilzadeh, 2021). For example, tieba.baidu has set up multiple discussion columns for chronic diseases, such as diabetes and hypertension. All these columns fall under the category of UGC, but a majority of users are at an inactive status, suggesting a lack of motive and intention to participate among them. This will finally impair the vitality for the community development. Hence, it is necessary to study users’ participation in OHCs from the perspective of knowledge acquisition and knowledge contribution.

### Users’ Participation

Users’ participation is described by many literatures as a psychological status. When the system can attract users’ attention, the user will be attracted by the system to obtain internal rewards (Johnston et al., 2013; Wu R.-Z. et al., 2021; Wu and Tian, 2021). Literatures of the early stage tend to associate participation with the network traffic theory. An eye-catching system can effectively attract users while interacting with it (Webster and Ahuja, 2006; Malinen, 2015). A research by Di Gangi and Wasko (2016) defined users’ participation in social media as “one kind of user psychology, which can not only ensure the improvement of users’ participation, but also bring benefits meaningful to individuals.”

Users’ participation is a critical index of OHCs (Bennett-Levy et al., 2021; Liu and Kong, 2021). OHCs can provide medical and health care service consumers with instant access to information, massive medical care information, and other medical and health care services (Loane et al., 2015; Lu et al., 2020). In addition to allowing users to search for health information, OHCs also facilitates information sharing (Yan et al., 2016). Individuals can easily share their medical and health care knowledge, experience and treatment plans, and post comments on healthcare products, drugs and doctors’ comments (Zhang et al., 2017a). In spite of the aforesaid advantages of OHCs as part of virtual communities, challenges facing OHCs in terms of users’ participation cannot be ignored (Zhao et al., 2021). To learn predictive factors of users’ participation can shed light on how to promote the sharing of online community information (Mirzaei and Esmaeilzadeh, 2021).

### Social Capital Theory

Social capital can reflect the total resources included in the relation network of an individual or a social network, and users can have social interaction based on resources possessed thereby to promote information exchange (Nahapiet and Ghoshal, 1998). Social capital is first use to study community relations, and people’s intention to share information and knowledge is subject to the impact of social capital (Wang T. et al., 2016). When social interaction is friendly, people are usually willing to share their knowledge and information (Chang and Chuang, 2011). Social capital theory holds that social resources can integrate some elements in the social network, and that social capital can not only achieve a consensus among individuals, but also force them to realize the shared objective (Petter et al., 2020).

Research suggests that social capital has three dimensions, including structural dimension, relational dimension and cognitive dimension, and that all these dimensions can control individual behaviors and interaction (Yang, 2021). First of all, structural capital stands for the contact model among individuals, which can reflect the users’ position in the social system and identify users’ ability to access resources (Zhou, 2016). In other words, structural capital can decide whom an individual can reach, and how the individual can reach the person, and can indicate the connection and availability of network friends (Zhou, 2019a). Whether network relation or any proper organization exists or is established among individuals constitutes the most important aspect of this dimension. Organizations of the kind reflect the relation models in terms of the scale, density, connection, hierarchical structure, etc. (Chiu et al., 2006). Proper organizations refer to the network that is set up for one shared purpose (Li and Ku, 2018).

Second, relational dimension can describe the trust among members in the communication process (Zhang et al., 2018). This dimension can reflect the attribute of the cooperation with other members and the quality of the interpersonal relationship, which is a kind of interpersonal relationship that is formed because of the interaction history among individuals (Yang et al., 2016). This concept focuses on the special relation among individuals, such as respect for friendship, which can affect their behaviors (Cheng et al., 2019). Besides, cognitive dimension refers to a shared understanding and opinion of something among individuals (Wang T. et al., 2016). The formation of the virtual community has a close bearing on the values and shared interests of individuals, the latter of which can be named as “shared vision” (Wang and Herrando, 2019). In a virtual community, the higher the similarity is between individuals, the more likely they will see the information interesting them, and the stronger their motive to seek the information will be (Zhou, 2018). At the same time, the virtual community is a platform for individuals to share similar experience and interest. The more the values and objectives are shared by individuals, the more confident the individuals will be to share opinions and comments (Ko, 2018).

### Social Support

Social support can reflect the experience of users to receive care, help and attention from the online community (Molinillo et al., 2018). Social support is provided from different dimensions. Liang et al. (2011) noticed that social support exchanged in social commerce includes informational support and emotional support. This measurement outcome has been applied to the follow-up research (Lin et al., 2015). Social support can include instrumental support and expressive support (Lin, 2011), which is similar to the informational support and emotional support, respectively. Prior research also points out that social support covers friendship, assessment and respect (Oh et al., 2014). Additionally, Yan and Tan (2014) pointed out that social support for online medical care community communication includes the informational support, emotional support and friendship.

Swift development of OHCs has benefited patients in many ways, enabling patients to have access to social support, which informational, emotional, etc. In this research, social support refers to an individual’s experience of getting support, care or help from a group. Informational support refers to providing suggestions, explanations or information that is conducive to problem-solving to help users resolve problems or organize their thinking to make a wise decision. Users’ information behaviors include sharing and exchange of health information, which can create favorable information sources for continuous value adding and communication of health information (Nambisan, 2011). Emotional support can fuel up sharing behaviors of community information (Zhang et al., 2017a). In this research, emotional support is defined as psychological support, such as sympathy, care or understanding, which can comfort users psychologically and help resolve problems indirectly. Hence, social support, in addition to enriching information sources, also warms individual’s heart (Lu et al., 2021). The favorable experience can enhance users’ willingness to seek social interaction with other users, while support providers can also obtain the sense of identity from interaction of the kind (Atanasova and Petric, 2019).

In the OHC, users hope that they can have access to not only valuable suggestions, but also emotional attention and encouragement (Li and Wang, 2018). Research has identified that social support is an important factor that affects OHC user behaviors (Yan et al., 2016; Liu et al., 2018). Therefore, this research includes social support into the model to verify its impact on social capital.

## Research Model and Research Hypotheses

### Research Model

**FIGURE 1:**
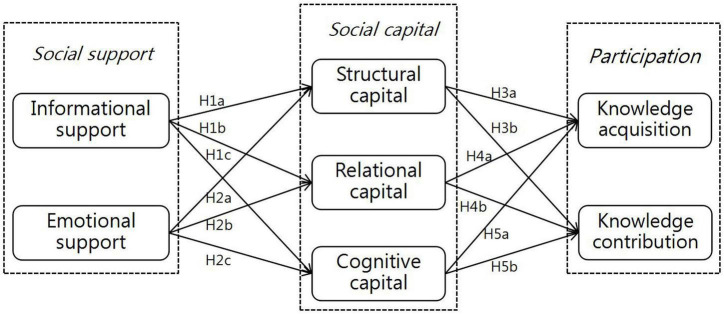
Research model.

Set against the background of OHCs and adopting social support as an antecedent variable, this research proposes a comprehensive model to verify the impact of three dimensions of social capital, including structural capital, relational capital and cognitive capital, on users’ participation (knowledge acquisition and knowledge contribution). Figure 1 displays the research model of this paper.

### Based on the Research Hypotheses of Social Support

Social support consists of informational support and emotional support (Liang et al., 2011), of which informational support can provide suggestions which can facilitate problem solution (Zhou, 2019a). When users acquire valuable health information from other members, they might perceive the ability and credibility of these members. This is conducive to developing users’ trust for OHCs, because the information can help them improve health. Besides, a frequent information interaction can enhance users’ familiarity with other members and strengthen their bilateral relation, such as social interaction and relation. Users might form the habit of social interaction in OHCs. Moreover, informational support can also affect relational capital, for it can promote users’ understanding of other members’ opinions. Last but not least, a frequent information interaction can contribute to the formation of a shared language among users. Users can create their commonly used terms and jargons through a frequent interaction, thus increasing their bilateral exchange. Therefore, we make the following hypotheses:

H1a. Informational support is positively related to structural capital.

H1b. Informational support is positively related to relational capital.

H1c. Informational support is positively related to cognitive capital.

Emotional support is the care, encouragement and sympathy expressed by other members (Zhou, 2019b). As an intermediate support, emotional support, though incapable of directly addressing problems, can otherwise warm users spiritually, thus stimulating users’ interaction with other members. So emotional support is critically important to users’ behavioral decision-making. Particularly to users of OHCs, they might be stuck in negative emotions, such as depression and anxiety (Yan and Tan, 2014). When users acquire emotional support from other members, they might perceive kindness from other members, and generate trust and recognition of OHCs (Xu et al., 2016). In the opinion, they have established the emotional link with their community. In addition, emotional support can promote the formation of a shared language among users, through which users can understand each other better. Thus, we suggest:

H2a. Emotional support is positively related to structural capital.

H2b. Emotional support is positively related to relational capital.

H2c. Emotional support is positively related to cognitive capital.

### Based on the Research Hypotheses of the Social Capital

Structural capital can show the social interaction among users (Luo et al., 2020). As one of major driving factors of user behaviors, social interaction can promote information and resource exchange among users (Mirzaei and Esmaeilzadeh, 2021). OHCs make ubiquitous interactions possible for users, which might increase users’ interaction frequency and duration, and promote social interaction among them (Zhao et al., 2012). So users with frequent social interaction with more frequently communicate with others about health information and share about medical care experience. In other words, a close connection can enhance users’ participation, for they hope that they can maintain a close connection with other users. Therefore, we suggest:

H3a. Structural capital is positively related to knowledge acquisition.

H3b. Structural capital is positively related to knowledge contribution.

Relational capital can reflect the quality of the relation of exchange, which includes trust and reciprocity (Hsu and Hung, 2013; Xu and Zhou, 2018). As an important factor that decides the quality of relations, trust is fostered through the frequent interaction among individuals (Karpenka et al., 2021). In the network environment, trust for others is a basic factor that drives users’ behavioral intention (Hsieh et al., 2021). Since the online community involves uncertainties and risks, such as the risk of information privacy leakage, it is necessary for users to establish trust with other community members so as to reduce the perceived risk (Yang and Lee, 2021). Besides, if there is a strong awareness of reciprocity, that is, if members believe resource exchange is reciprocal, they will show more intention to participate (Xu and Zhou, 2018). In other words, users disclosing information are also expecting information. Otherwise, they might deem the exchange as unfair and stop participating in the exchange. Thus, we suggest:

H4a. Relational capital is positively related to knowledge acquisition.

H4b. Relational capital is positively related to knowledge contribution.

Cognitive capital is a mirror of shared language among users (Yang, 2021). A frequent interaction can increase users’ mutual understanding and help them form a shared language, such as common terms and jargons (Zhao et al., 2012). In addition to enhancing the shared perception and understanding among individuals, shared language can also reduce every individual’s cognitive barriers (Xu and Zhou, 2018). This can improve the communication efficiency and promote users’ participation. Because of the difficulty facing users’ entering in the information on the mobile terminals, a shared language is critical to their communication and interaction. Thus,

H5a. Cognitive capital is positively related o knowledge acquisition.

H5b. Cognitive capital is positively related to knowledge contribution.

## Materials and Methods

The research model covers seven factors, each of which is measured by three or four items. In order to improve the content validity, all items are adapted from the existing literatures. The questionnaire on structural capital, relational capital, and cognitive capital is adapted from that of Zhao et al. (2012) and Xu and Zhou (2018). The questionnaire on knowledge acquisition and knowledge contribution is adapted from that of Zhou (2020) and Chen et al. (2020). The questionnaire on informational support and emotional support is adapted from that of Liang et al. (2011) and Zhou (2019a). The unit of analysis focused on the individual and the responses were measured using a 5-point Likert scale on an interval level ranging from “strongly agree” to “strongly disagree” (refer to Appendix A).

Based on a comprehensive review of research findings by scholars at home and abroad, and in order to ensure all items to adapt to the OHC environment, we invite six experts and professors from the chronic disease and information system management field to assess the selection of questions and whether bias exists in these questions. Finally, items consistent with the OHC environment are chosen. After that, in order to test the instrument, a preliminary test is conducted on 20 users with OHC use experience. According to pre-survey results and suggestions of professors, the wording of questions is modified moderately under the prerequisite of not changing the connotation of questions so as to improve the clarity and comprehensibility of questions. At last, the questionnaire survey is designed. The final version of these items is translated by the professional translator independently into Chinese, which is then translated by another translator into English again to ensure the equivalence of translation.

Data of this research are collected through online surveys. Considering the dramatic difference potentially existing in the demand of chronic disease patients and non-chronic disease patients for medical information, and to more accurately predict OHC members’ participating behaviors, this research adopts chronic disease patients as research samples to measure participating behaviors of OHC members. We post survey links on platforms, including WeChat, Weibo, Baidu, etc. We have also invited users with the OHC use experience to fill in the questionnaire. The survey lasted for 4 months. Finally, 1309 copies of questionnaire were collected from 20 March 2021 to 20 July 2021. We also scrutinized all responses and dropped 32 that had too many missing values. As a result, we obtained 1277 valid responses. Table 1 shows the demographic information of the respondents.

**TABLE 1:** Sample characteristics.

Variable		Number	Percentage
Gender	Male	741	58%
	Female	536	42%
Age	<20	83	6.5%
	21–30	171	13.4%
	31–40	503	39.4%
	41–50	309	24.2%
	>50	211	16.5%
Education	Middle school or below	34	2.7%
	Senior high school	102	8.2%
	Junior college	177	13.2%
	Undergraduate	892	70.1%
	Postgraduate or above	72	5.8%
Usage history	Baidu post bar	387	30.3%
	Good doctor	168	13.2%
	Zhihu	267	20.9%
	Douban	134	10.5%
	Weibo	174	13.6%
	Others	147	11.5%
Experience	Yes	1277	100%

## Results

Structural equation modeling (SEM) together with SPSS 23.0 and AMOS 23.0 is adopted to analyze the research data.

### Measurement Model

First, we conducted a reliability and convergent validity. As listed in Table 2, each loadings are larger than 0.7. Dijkstra–Henseler’s rho and Jöreskog’s rho (composite reliability) exceeds 0.7, and each average variance extracted (AVE) exceeds 0.5. In addition, all Cronbach alpha values are larger than 0.7. This indicated the excellent reliability and convergent validity (Hair et al., 2010; Henseler, 2010).

**TABLE 2:** Standardized item loadings, AVE, alpha, and rho values.

Factor	Item	Standardized loading	Alpha	AVE	Jöreskog’s rho (pc)	Dijkstra–Henseler’s rho (pA)
Informational support (IS)	IS1	0.804	0.828	0.621	0.831	0.829
	IS2	0.734				
	IS3	0.824				
Emotional support (ES)	ES1	0.807	0.814	0.595	0.815	0.814
	ES2	0.745				
	ES3	0.760				
Structural capital (SC)	SC1	0.846	0.873	0.636	0.876	0.874
	SC2	0.836				
	SC3	0.811				
	SC4	0.697				
Relational capital (RC)	RC1	0.715	0.828	0.549	0.829	0.829
	RC2	0.768				
	RC3	0.732				
	RC4	0.747				
Cognitive capital (CC)	CC1	0.755	0.818	0.530	0.818	0.818
	CC2	0.709				
	CC3	0.749				
	CC4	0.698				
Knowledge acquisition (KA)	KA1	0.698	0.814	0.523	0.814	0.814
	KA2	0.740				
	KA3	0.725				
	KA4	0.729				
Knowledge contribution (KC)	KC1	0.738	0.842	0.574	0.843	0.842
	KC2	0.729				
	KC3	0.768				
	KC4	0.794				

Table 3 lists the square root of AVE (shown as bold at diagonal) and factor correlation coefficients, for each factor, the square root of AVE is significantly larger than its correlation coefficients with other factors, suggesting excellent discriminant validity (Hair et al., 2010).

**TABLE 3:** Matrix of correlation constructs and discriminant validity.

	IS	ES	SC	RC	CC	KA	KC
IS	**0.788**						
ES	0.429	**0.77**					
SC	0.144	0.2	**0.797**				
RC	0.499	0.494	0.163	**0.740**			
CC	0.426	0.418	0.152	0.77	**0.728**		
KA	0.383	0.423	0.154	0.663	0.719	**0.723**	
KC	0.478	0.393	0.149	0.795	0.708	0.605	**0.757**

*The square root of AVE (shown as bold at diagonal) and factor correlation coefficients.*

### Structural Model

We adopted structural equation modeling software AOMS 23.0 to estimate the structural model. Table 4 lists the recommended value (Hair et al., 2010) and actual values of structural model fit, all fit indices have better actual values than the recommended values.

**TABLE 4:** Fit indicators of the structural models.

Model fit indices	χ^2^/df	NFI	CFI	GFI	IFI	AGFI	RMSEA
Recommended value	1–3	>0.90	>0.90	>0.90	>0.90	>0.80	<0.08
Actual value	2.969	0.946	0.964	0.953	0.964	0.942	0.039

*χ^2^/df Chi-squared divided by degrees of freedom.*

*NFI, normed fit index; AGFI, adjusted goodness-of-fit index; CFI, comparative fit index; IFI, incremental fit index; GFI, goodness-of-fit index; RMSEA, root mean square error of approximation.*

**FIGURE 2:**
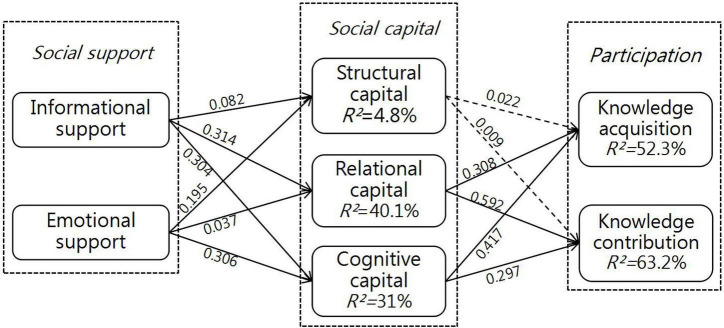
Structural model results. *p < 0.05; **p < 0.01; ***p < 0.001; the dotted line represents the insignificant path.

Table 5 presents the results. Except H3a and H3b, other hypotheses were supported. Factors performance expectancy have high loadings on the continuous usage intention. In Figure 2, the explained variance of, structural capital, relational capital, cognitive capital, knowledge acquisition, and knowledge contribution is 4.8, 40.1, 31, 52.3, and 63.2%, respectively.

**TABLE 5:** Results of the hypotheses tests.

Path		Estimate	SE	CR (*p*-value)	Results
H1a	Informational support → structural capital	0.082	0.040	2.058 (0.04)	Supported
H1b	Informational support → relational capital	0.314	0.029	10.995 (***)	Supported
H1c	Informational support → cognitive capital	0.304	0.033	9.323 (***)	Supported
H2a	Emotional support → structural capital	0.195	0.042	4.670 (***)	Supported
H2b	Emotional support → relational capital	0.307	0.03	10.354 (***)	Supported
H2c	Emotional support → cognitive capital	0.306	0.034	9.019 (***)	Supported
H3a	Structural capital → knowledge acquisition	0.022	0.018	1.204 (0.229)	Not supported
H3b	Structural capital → knowledge contribution	0.009	0.018	0.526 (0.599)	Not supported
H4a	Relational capital → knowledge acquisition	0.308	0.030	10.363 (***)	Supported
H4b	Relational capital → knowledge contribution	0.592	0.035	16.814 (***)	Supported
H5a	Cognitive capital → knowledge acquisition	0.417	0.029	14.218 (***)	Supported
H5b	Cognitive capital → knowledge contribution	0.297	0.026	11.312 (***)	Supported

***p < 0.001.

## Discussion

As the awareness of health is strengthening among Chinese nationals, online medical care information has gained increasing popularity, which has driven the development of OHCs. A critical factor that affects the development of OHCs is users’ participation. Pitifully, the motive for users to participate in OHCs is often lacking. Therefore, this research examines decisive factors that influence OHC users’ participation from the perspective of social capital theory.

As presented in Table 5 and Figure 2, H1 is fully substantiated. Results show that informational support has a significant impact on structural capital (β = 0.082, *p* < 0.05), relational capital (β = 0.314, *p* < 0.01), and cognitive capital (β = 0.304, *p* < 0.01). This finding coincides with that of Zhou (2019b) and suggests that users are more willing to adopt opinions of other community members, if they can acquire valuable information from the interaction. This also indicates that users who are willing to use OHCs are all pragmatic. For example, users suffering from certain disease aim at seeking valuable information. If professional knowledge acquired from other members can give some help to alleviate their symptoms, then users will be more willing to share their knowledge (such as experience) with patients suffering from the same disease. Hence, informational support can show the value of OHCs, and enhance users’ sense of belonging and cohesion, which can then promote users’ participation.

Similarly, H2 is fully substantiated. Emotional support has a significant impact on structural capital (β = 0.195, *p* < 0.01), relational capital (β = 0.307, *p* < 0.01), and cognitive capital (β = 0.306, *p* < 0.01). This finding shows good agreement with that of Zhou (2020). This suggests that emotional support given by other OHC members can comfort users, because a majority of them share the experience of their disease. The comfort from users with the same or similar experience can easily strike a chord in each other’s heart, and the comforted user will generate a sense of belonging toward the community deep down inside. Therefore, emotional support can help users perceive more warmth, who will them internalize the community values into their code of conduct. The comfort, encouragement and care provided by emotional support can effectively ease users’ negative emotions (such as anxiety and depression), which can boost users’ establishment of structural capital, relational capital, and cognitive capital of OHCs.

Relational capital can directly exert a positive impact on knowledge acquisition (β = 0.308, *p* < 0.01) and knowledge contribution (β = 0.592, *p* < 0.01). This finding shed light on the impact of other community members’ trust and reciprocity on users’ participation in OHCs. Users are willing to accept reliable and reciprocal suggestions and recommendations from other OHC members, and share their own knowledge of health. Participating in OHCs might be deemed as a risky behavior. Thereby, trust and reciprocity have been decisive factors that can directly predict users’ participation in OHCs. This research result is consistent with prior research finding. For example, Luo et al. (2020) discovered a positive correlation between trust and trading intention in e-commerce.

Like relational capital, cognitive capital can directly exert a positive impact on knowledge acquisition (β = 0.0417, *p* < 0.01) and knowledge contribution (β = 0.297, *p* < 0.01). This finding is consistent with that of Chen et al. (2020), and also indicates that users’ participatory behaviors in OHCs are decided not only by trust and reciprocity, but also by the shared language among community members. This suffices to show the importance of a shared language in OHC. For example, the public terms and jargons can promote communication and interaction among users, and improve their communication efficiency.

At last, the impact of structural capital on knowledge acquisition (β = 0.022, *p* > 0.1) and knowledge contribution (β = 0.009, *p* > 0.1) is not significant. This suggests that where there is a strong interaction between one user and other members, they might establish a close connection and deepen their participation, which is contradictory to our prediction. The contradiction is probably caused by OHCs as a platform for commenting, adding likes to and searching for professional knowledge online. Compared with the real-life communities, virtual communities have one striking defect, that is, the fragile connection among their members, which can negatively influence user behaviors. This research result is consistent with that of Xu and Zhou (2018) and Yang (2021).

## Theoretical and Practical Implications

This study proposed an extended information system success model to gain a deeper understanding of the factors affecting continued intentions to use a mobile workplace. The results suggested that the system quality, information quality, and collaboration quality have all made significant contributions to promoting performance expectancy and satisfaction. The performance expectancy and satisfaction could in turn boost the continuance intention. The empirical results also provided solid evidence for the good explaining power of the informational support (IS) success model for this model. This substantiated its validity in predicting users’ continued intention to use mobile workplaces. Therefore, the IS success theory was a favorable model to explain the mobile workplace. Besides this, we combined the IS success model, collaboration quality, and performance expectancy to verify its feasibility. By emphasizing the feasibility of extending the publicly acknowledged model theory, this paper could provide an enriched insight and contribution to the literature on information systems. To researchers, this research could provide a solid foundation for the further improvement of the IS success theory and be regarded as a springboard for future research. We are convinced that this model could be further tested in different countries and among different age groups and information systems.

### Theoretical Implications

First of all, prior research has extensively tested consumers’ behavior intentions using social capital theory. For example, Yang (2021) adopted social capital theory to verify consumers’ purchase intentions in social commerce. Another example is the verification of the impact of social capital on consumer value and consumers’ loyalty in online outshopping. Nevertheless, little research attention has been paid to the correlation between social capital and OHC users’ participation. Considering the above research gap, this research adopts social support theory as an antecedent variable based on social capital theory to verify OHC users’ participation.

Second, in order to clarify the complex correlations of different dimensions of social capital theory, this research explores the impact of different dimensions of social capital theory on users’ participation. Specifically, relational capital and cognitive capital have a significantly positive impact on knowledge acquisition and knowledge contribution, while knowledge acquisition and knowledge contribution are free from the impact of structural capital. This finding verifies that three dimensions of social capital theory have different functioning mechanisms under different research backgrounds. Under the C2C background Huang et al. (2015) found that social capital can cause different changes along with different roles of individuals. Chiu et al. (2006) observed that the impact of social capital on the quantity and quality of knowledge shared is varied. Hence, our research findings have enriched the understanding of how the three dimensions of social capital, namely structural dimension, relational dimension, and cognitive dimension, influence users’ participation.

### Managerial Implications

This research can also provide some managerial implications. Research results show that OHC managers should encourage community members to develop a strong social capital. It is observed that social support can significantly influence social capital. Therefore, OHC managers need to create a supportive atmosphere to develop social capital and promote users’ participation in OHCs. On the other hand, relational capital and cognitive capital have a significant impact on knowledge acquisition and knowledge contribution. In other words, trust for colleagues, reciprocity and shared language are major decisive factors of users’ participation. This means that only when users can acquire valid information from OHCs will they share their own knowledge. This necessitates the establishment of regulations to encourage users to communicate with each other honestly and sincerely, which can benefit the creation of a reciprocal atmosphere. OHC managers can encourage users to exchange social support with other members *via* rewards, such as integrated points and rewards. They can also choose some critical opinion leaders as examples. Besides, OHCs should take measures to promote the formation of a shared language. They can analyze user texts to acquire commonly used terms and jargons, and list these terms for users’ reference. Service providers should also build a credible environment for users and develop the recognition between them and the community.

## Limitations

Our research limitations suggest the necessity of future research. First, the correlation between the OHC users’ participation and social capital might be a dynamic process, while this text adopts the cross-sectional data to test the model. Therefore, the future research can examine dynamic changes of user behaviors.

Second, OHCs can be divided into different types. User behaviors from different types of communities can be varied. Hence, the future research can pursue a comparative analysis of different types of OHC user behaviors, such as examining the difference of users’ participation in diabetes communities and hypertension communities.

## Data Availability Statement

The original contributions presented in this study are included in the article/supplementary material, further inquiries can be directed to the corresponding author.

## Ethics Statement

The studies involving human participants were reviewed and approved by the Jiaxing University. Written informed consent for participation was not required for this study in accordance with the national legislation and the institutional requirements.

## Author Contributions

X-FT contributed to the research design, empirical analysis, and manuscript writing. R-ZW conducted the methodology and research design, and developed the original idea for the study. Both authors read and approved the final manuscript.

## Conflict of Interest

The authors declare that the research was conducted in the absence of any commercial or financial relationships that could be construed as a potential conflict of interest.

## Publisher’s Note

All claims expressed in this article are solely those of the authors and do not necessarily represent those of their affiliated organizations, or those of the publisher, the editors and the reviewers. Any product that may be evaluated in this article, or claim that may be made by its manufacturer, is not guaranteed or endorsed by the publisher.

## Appendix

**Appendix A:** The study’s measurement items.

Dimensions	Texts of items
Structural capital (Zhao et al., 2012; Xu and Zhou, 2018)	I maintain close social relationships with other members on OHCs.
	I spend a lot of time interacting with other members on OHCs.
	I know some members on OHCs at a personal level.
	I have frequent communication with other members on OHCs.
Relational capital (Zhao et al., 2012; Xu and Zhou, 2018)	The relationship is characterized by mutual respect between me and other embers on OHCs.
	The relationship is characterized by personal friendship between me and other members on OHCs.
	The relationship is characterized by mutual trust between me and other members on OHCs.
	The relationship is characterized by high reciprocity between me and other members on OHCs.
Cognitive capital (Zhao et al., 2012; Xu and Zhou, 2018)	When interacting with other members on OHCs, we use common terms or jargon.
	I feel members in the OHCs have values similar to mine.
	When communicating with other members on OHCs, we use understandable narrative forms.
	I feel members in the OHCs have experiences similar to mine.
Knowledge acquisition (Chen et al., 2020; Zhou, 2020)	I often visit this community to acquire knowledge on a particular health topic.
	I come to the OHCs to get information on a particular health subject.
	When faced with health difficulties, I will seek help in the community.
	I will consider coming to the OHCs to seek related information when I need to know a particular health topic.
Knowledge contribution (Chen et al., 2020; Zhou, 2020)	I’d like to share my commentary with other members of the OHCs.
	I visit this community to share my health skills and experience with other members.
	I will disclose more health information in the community in future.
	I visit this community to give other members information I know about a particular health subject.
Informational support (Liang et al., 2011; Zhou, 2019a)	In the community, some people would offer suggestions when I needed help.
	When I encountered health problems, some people in the community would give me information to help me overcome the problem.
	When faced with health difficulties, some people in the community would help me discover the cause and provide me with suggestions.
Emotional support (Liang et al., 2011; Zhou, 2019a)	When faced with health difficulties, some people in the community comforted and encouraged me.
	When faced with health difficulties, some people in the community listened to me talk about my private feelings.
	When faced with health difficulties, some people in the community expressed interest and concern in my well-being.
